# Dental status, dental treatment procedures and radiotherapy as risk factors for infected osteoradionecrosis (IORN) in patients with oral cancer – a comparison of two 10 years’ observation periods

**DOI:** 10.1186/2193-1801-3-263

**Published:** 2014-05-23

**Authors:** Marcus Niewald, Kristina Mang, Oliver Barbie, Jochen Fleckenstein, Henrik Holtmann, Wolfgang J Spitzer, Christian Rübe

**Affiliations:** Department of Radiotherapy and Radiooncology, Saarland University Medical Center, Building 6.5, Kirrberger Strasse 100, D-66424 Homburg/Saar, Germany; Dental Practice, Duisburg, Germany; Dental Practice, Lebach, Germany; Department of Oral and Maxillofacial Surgery, Saarland University Medical Center, Homburg/Saar, Germany; Department of Cranio-and-Maxillo Facial Surgery, Düsseldorf University Medical School, Düsseldorf, Germany

**Keywords:** Oral cancer, Radiotherapy, Infected osteoradionecrosis, Dental status

## Abstract

**Objectives:**

Dental status, dental treatment procedures and radiotherapy dosage as potential risk factors for an infected osteoradionecrosis (IORN) in patients with oral cancers: Retrospective evaluation of 204 patients treated in two observation periods of approximately ten years each.

**Patients and methods:**

In group A, 90 patients were treated in the years 1993-2003, in group B 114 patients in the years 1983-1992 (data in brackets). All patients had histopathologically proven squamous cell cancers, mainly UICC stages III and IV. 70% (85%, n.s.) had undergone surgery before radiotherapy. All patients were referred to the oral and maxillofacial surgeon for dental rehabilitation before further treatment.

Radiotherapy was performed using a 3D-conformal technique with 4-6MV photons of a linear accelerator (Co-60 device up to 1987). The majority of patients were treated using conventional fractionation with total doses of 60-70 Gy in daily fractions of 2 Gy. Additionally, in group A, hyperfractionation was used applying a total dose of 72 Gy in fractions of 1.2 Gy twice daily (time interval > 6 hours). In group B, a similar schedule was used up to a total dose of 82.8 Gy (time interval 4-6 hours). 14 (0) patients had radiochemotherapy simultaneously. After therapy, the patients were seen regularly by the radiooncologist and – if necessary – by the oral and maxillofacial surgeon. The duration of follow-up was 3.64 years (5 years, p = 0.004).

**Results:**

Before radiotherapy, the dental health status was very poor. On average, 21.5 (21.2, n.s.) teeth were missing. Further 2.04 teeth (2.33, n.s.) were carious, 1.4 (0.3, p = 0.002) destroyed.

Extractions were necessary in 3.6 teeth (5.8, p = 0.008), conserving treatment in 0.4 (0.1, p = 0.008) teeth. After dental treatment, 6.30 (4.8, n.s.) teeth remained.

IORN was diagnosed after conventionally fractionated radiotherapy in 15% (11%, n.s.), after hyperfractionation in 0% (34%, p = 0.01).

**Conclusion:**

Within more than 20 years there was no improvement in dental status of oral cancer patients. Extensive dental treatment procedures remained necessary. There was an impressive reduction of the IORN frequency in patients treated in a hyperfractionated manner probably resulting from a dose reduction and an extension of the interfraction time.

## Background

Infected osteoradionecrosis (IORN) is still one of the major problems after radiotherapy for neoplasms in the oral cavity. The chewing and swallowing functions of the patients are impaired, long-lasting conservative and surgical interventions may become necessary. In the last decades, there was an ample discussion about potential risk factors for the development of IORN. Besides the kind of surgical procedures, dosage and fractionation of radiotherapy and the simultaneous application of chemotherapy, the patients’ dental status before treatment and the extent of dental treatment procedures were regarded significant.

Thus, more than 20 years ago, the departments of Radiotherapy and of Oral and Maxillofacial Surgery of the Saarland University Medical School together started a dental examination and rehabilitation program with the aim to prevent IORN as far as possible. All patients referred for radiotherapy for cancer in the oral cavity were examined by the oral and maxillofacial surgeon, the exact dental findings were recorded and – if necessary, specific rehabilitation procedures were performed. Radiotherapy was started only after approval by the Oral and maxillofacial surgeon (OMF surgeon). After therapy, the patients were seen regularly by the radiooncologist, diagnosis and treatment of IORN were performed by the oral and maxillofacial surgeon.

After completion of two theses (Barbie 1997; Mang 2011), published in (Niewald et al. 1996; Niewald et al. 2013) each analyzing a ten years’ period of radiotherapy for oral cancer one after the other, we now had the unique possibility to reanalyze and to compare the data obtained in this very long observation period in terms of dental findings, dental rehabilitation procedures and the frequency of IORN.

## Methods

Two groups of patients who had undergone radiotherapy for neoplasms of the oral cavity have been reanalyzed retrospectively:

Group A consists of 90 consecutive patients having been treated in the years 1993-2003.

Group B consists of 114 consecutive patients having been treated in the years 1983-1992 (data in brackets).

All patients suffered from squamous cell carcinoma of the oral cavity mainly in stages III and IV according to the Union Internationale contre le Cancer-(UICC)-definition, one patient had a local recurrence but had not been irradiated before. Patients with treated local or regional recurrences, distant metastases or with insufficient data were excluded. In group B, it seemed impossible to actualize the follow-up data, so that the data from the former analysis were taken. For this reason, no comparison of oncological results was attempted. In order to improve comparability, the inclusion criteria mentioned above were applied to both groups retrospectively which lead to the exclusion of several patients and a complete re-analysis of the data.

70% of the patients in group A (85%, n.s.) had undergone surgery for the primary tumor and the regional lymph node regions. After surgery or after biopsy all patients were referred to the oral and maxillofacial surgeon for assessment of the dental status including an meticulous clinical and x-ray examination. The dental treatment procedures were performed as early as possible with a minimal time interval of 7-10 days from the last procedure to the beginning of radiotherapy. All dental extractions were performed according to a written protocol under “special care” (primary tissue closure, perioperative antibiotics for 7-10 days beginning one day before surgery). In the nineties all patients were advised not to wear their dental prostheses up to 6-12 months after radiotherapy (today until complete healing of mucositis) (Curi and Dib 1997; Reuther et al. 2003). Radiotherapy was started after complete healing of the gingival wounds and thus approval by the OMF surgeon.

After production of a face mask for fixation, the computerized tomography for radiotherapy planning was performed, and the two- dimensional (up to 2000) or three-dimensional dose distribution was computed after target volume delineation. Radiotherapy was applied using 4-6MV photons (electrons for level V lymph node region) of a linear accelerator; a 60-Cobalt machine was in use additionally until 1987. In the majority of patients (n = 73 in group A, n = 74 in group B), conventionally fractionated radiotherapy was applied with total doses of 60-70 Gy (details see Table 1) in daily single fractions of 2 Gy. Furthermore, different hyperfractionated schedules were performed in both groups: in group A (n = 46) a total dose of 72 Gy was applied in single doses of 1.2 Gy twice daily (interfraction interval > =6 hours) to patients with formerly untreated tumors, in group B (n = 41) a total dose of 82.8 Gy in single doses of 1.2 Gy twice daily (interfraction interval 4-6 hours) was applied to patients with untreated and with resected tumors. Two patients in group A have been treated in a different manner (one with 1.4 Gy twice daily, another with a single dose of 3.0 Gy once daily).

**Table 1:** **Biographical and oncological data**

Item		Group A	Group B	Remarks
Mean age (years)		57.1	54.6	n.s.
Mean Karnofsky performance Index		7.8	7.5	n.s.
Follow-up (years)		4.1	5.0	p = 0.004
T-stage	T1	14 (16%)	20 (18%)	n.s.
	T2	33 (37%)	49 (43%)	
	T3	8 (9%)	19 (16%)	
	T4	35 (38%)	26 (23%)	
N-stage	N0	20 (22%)	46 (40%)	n.s.
	N1	23 (26%)	24 (21%)	
	N2	47 (52%)	30 (26%)	
	N3	0	14 (13%)	
UICC stage	I	6 ( 7%)	10 ( 9%)	n.s.
	II	7 (8%)	22 (19%)	
	III	19 (21%)	26 (23%)	
	IV	58 (64%)	56 (49%)	
Pre-treatment	None	24 (27%)	17 (15%)	p < 0.001
	Surgery	66 (73%)	97 (85%)	
Total dose (Gy)	Conventional fractionation	N = 75	N = 73	p < 0.001
		30Gy (1)	2Gy (1)	
		36Gy (1)	8Gy (1)	
		50Gy (7)	36Gy (1)	
		58Gy (2)	44Gy (1)	
		60Gy (32)	56Gy (1)	
		64Gy (9)	60Gy (31)	
		70Gy (23)	62Gy (1)	
			66Gy (1)	
			70Gy (31)	
			72Gy (2)	
			76Gy (1)	
			80Gy (1)	
Total dose (Gy)	Hyperfractionation	N = 15	N = 41	
		55.8Gy (1)	13.2Gy (1)	
		70.8Gy (1)	81.6Gy (2)	
		72.0Gy (11)	82.8Gy (35)	
		72.8Gy (1)	85.2Gy (1)	
		76.8Gy (1)	85.5Gy (1)	
			87.8Gy (1)	
Daily fraction	Conventional fractionation	2.0 Gy (74)	2.0Gy (73)	p = 0.0012
		3.0 Gy (1)		
	Hyperfractionation	1.2 Gy (14)	1.2 Gy (41)	
		1.4 Gy (1)		
Simultaneous chemotherapy		14	0	p < 0.001

14(0) patients received chemotherapy consisting of cis-platinum and 5-FU simultaneously due to their unfavourable tumour and nodal stage. No patients with chemotherapy were excluded from the evalution. During therapy, the patients received dental care by the local dental colleagues. Fluoridation was used according to dental advice. Splints were not normally used because of unfavourable experience of patients with aggravating radiation mucositis by applying fluoride jelly to the gingiva using these splints.

After radiotherapy, the patients were examined for locoregional result and possible side effects in the Department of Radiotherapy and Radiooncology. Dental follow-up was performed by their local dentists. Consequently, detailed data about this phase are not available. Patients with a suspicion of IORN were referred to the Dept. of Oral and Maxillofacial Surgery for further diagnosis and treatment.

The mean duration of follow-up was 3.64 years (5 years, p = 0.004).

Infected osteoradionecrosis was minimally diagnosed when necrosis of the gingiva on top of the eroded bone became visible as infected mucosal ulcers with eroded mandibular bone underneath according to grade 2 or higher of the classification published by Schwartz et al. (Schwartz and Kagan 2002). Patients with manifest IORN were treated by the oral and maxillofacial surgeon in cooperation with the local dentist.

The patients’ data were collected from the records in the Departments of Radiotherapy and Radiooncology and Oral and Maxillofacial Surgery. All (panoramic x-ray) examinations available have been reviewed, thus we are quite sure that a potential local recurrence has not been misdiagnosed as an IORN. Furthermore, standardized questionnaires were mailed to the patients’ general medical practitioners and general dentists as well as the local authorities five times within the observation period in order to get additional data about freedom of local or regional recurrence, survival or the onset of IORN.

All data were entered into a medical databank (Medlog™, Parox, Muenster, Germany). Frequency distributions, means and standard deviations were computed. The groups were compared using the t-test (means) and the Kruskal-Wallace test (distributions). Overall survival and occurrence of IORN over time were computed using the Kaplan-Meier estimate, the comparison of the groups was performed using the Mantel-Haenszel test. Prognostic parameters for IORN were analyzed univariately by comparison of means and distributions in a group containing the patients with IORN compared to another group with the patients who never experienced IORN using the t-test, u-test and chi-square test in the appropriate variables. Multivariate search for independent prognostic factors was performed by logistic regression.

Detailed biographical and oncological data have been summarized in Table 1.

All patients had given their written informed consent before dental examination and treatment as well as radiotherapy. The approval by the local ethics committee was dispensable due to the retrospective nature of this evaluation. This research is in compliance with the Declaration of Helsinki in its actual version.

## Results

### General remarks

In group A, up to July 2013, 58 patients were dead with a mean follow-up of 2.4 [0-8.8] years. The patients known to be alive were seen irregularly, the most recent information resulted from questionnaires, nearly all patients were lost to follow-up after on average 7.4 [0-15] years.

In group B, 77/114 patients were dead with a mean follow-up of 3.4 [0-11.7] years. The patients known alive were lost to follow-up after on average 8.5 [4.3-13.3] years.

### Dental findings before radiotherapy

The patients’ dental status was generally poor. On average 10.1 (10.8) teeth were found present at the time of initial dental examination. For most of the criteria there was no statistically significant difference between the groups. However, we found significantly more destroyed teeth (1.4 vs. 0.3 teeth, p = 0.002) in the more recent patient collective, Furthermore there were more roots filled incompletely (0.3 vs. 0.1 teeth, p = 0.006). However, avital teeth were found less frequently in group A (0.5 vs. 1.0 teeth, p = 0.023). Chronic periodontitis with less to moderate attachment loss was found less frequently (p = 0.01) whereas chronic periodontitis with severe attachment loss was seen more frequently (p < 0.001) in group A compared to group B. Detailed data have been depicted in Table 2.

**Table 2:** **Dental findings before radiotherapy**

Teeth (mean values, n=)	Group A	Group B	Comparison:	Data available from n patients
Absent	22.0	21.2	n.s.	204
Present	10.1	10.8	n.s.	204
Carious	2.0	2.3	n.s.	198
Deeply carious destroyed	1.4	0.3	p = 0.002	200
Loose	1.6	2.2	n.s.	197
Root remainders	0.3	0.4	n.s.	202
Devital	0.5	1.0	p = 0.023	200
Roots – filled completely	0.2	0.1	n.s.	198
Roots – filled incompletely	0.3	0.1	p = 0.006	199
Apical periodontitis	0.3	0.2	n.s.	200
Dentogenic cysts	0.2	0.1	n.s.	199
Retained teeth	0.2	0.1	n.s.	198
Superficial marginal periodontitis (patients)				
- Localized	7 (8%)	33 (29%)	p = 0.002	199
- General	10 (11%)	16 (14%)		
Profound marginal periodontitis (patients)				
- Localized	12 (14%)	25 (22%))	p = 0.002	201
- General	35 (40%)	14 (12%)		

Superficial marginal periodontitis: chronic periodontitis with less to moderate attatchment loss.

Profound marginal periodontitis: chronic periodontitis with severe attatchment loss.

We can summarize that dental status in these special patients has hardly changed over the decades. Data concerning dental biofilm or the use of dental prostheses had not been collected in group B, thus a comparison could not be performed.

### Dental rehabilitation procedures

In the majority of criteria, the extent of dental rehabilitation procedures was identical in both groups. Tooth extractions were found more frequently in group B (3.7 vs. 5.8, p = 0.008) whereas conserving treatment was performed more frequently in Group A (0.6 vs. 0.1, p = 0.008). Detailed data are summarized in Table 3.

**Table 3:** **Dental rehabilitation procedures**

Teeth (mean values, n=)	Group A	Group B	Comparison	Data available from n patients
Endodontic treatment	0.05	0.03	n.s.	200
Removal of root remainders	0.2	0.4	n.s.	200
Tooth extraction	3.7	5.8	p = 0.008	202
Conserving treatment	0.6	0.1	p = 0.008	200
Cystectomy	0.09	0.05	n.s.	199
Surgical removal	0.3	0.2	n.s.	201
Healthy teeth remaining after dental rehabilitation	6.2	4.8	n.s.	201

### Frequency and risk factors of infected osteoradionecrosis (IORN)

IORN was found in the corpus region of the mandible in 11/90 patients (12%) of group A and 22/114 patients (19%) of group B (n.s.). The one-year prevalence was 5%, the two- and three-year prevalence 15%. A subgroup analysis dividing the collectives into two groups each with the patients having been treated with conventional fractionation or with hyperfractionation yielded the following results:

After conventional fractionation IORN was found in group A in 11/74 patients (15%), in group B in 8/73 patients (11%, differences n.s.). After hyperfractionation, IORN was not diagnosed in group A wheras it was observed in 14/41 patients (34%) in group B (p = 0.01).

**Figure 1:**
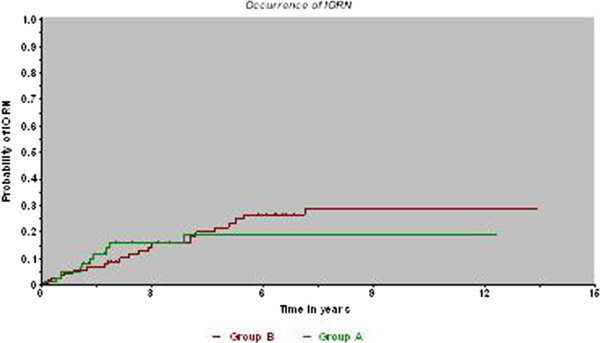
**Development of IORN over time (Kaplan-Meier estimate)**.

The Kaplan-Meier estimate showed that IORN normally occurred in the first two years in group A (first five years in group B) after radiotherapy (differences n.s.), after that time the risk remained stable (Figure 1). The subgroup analysis mentioned above resulted in identical curves for patients irradiated conventionally and highly different (but not statistically significant) curves after hyperfractionation.

The search for prognostically significant factors for the occurrence of IORN was performed using the whole patient collective consisting of 204 patients. The number of carious teeth, the N-stage, the total dose, the size of the daily fractions and the BED2 (Biologically effective dose 2 Gy) were found prognostically significant in univariate analysis. These factors were entered into the multivariate analysis where solely the number of carious teeth was found significant (details are depicted in Table 4). The multivariate analysis showed the number of carious teeth a nearly significant prognostic factor, furthermore, the fractionation was found to be trendwise significant. All further factors mentioned in Tables 1 and 2 tested univariately were found insignificant. Further details are depicted in Table 4.

**Table 4:** **Prognostic factors for the occurrence of IORN (n = 204)**

Univariate analysis		
Teeth	p=	Remarks
Dental status before starting radiotherapy		
Absent	0.110	
Present	0.107	
Carious	0.026	Significant
Deeply carious destroyed	0.202	
Loose	0.104	
Root remainders	0.595	
Devital	0.287	
Roots – filled completely	0.949	
Roots – filled incompletely	0.789	
Apical periodontitis	0.888	
Cysts	0.392	
Retained	0.1620	
Conservative treatment possible	0.430	
No conservative treatment possible	0.179	
Filled	0.751	
Not sufficiently filled teeth	0.549	
Teeth with not sufficient crowns	0.968	
Item		
Chronic periodontitis with less to moderate attatchment loss	0.418	
Localized		
General		
Chronic periodontitis with severe attatchment loss	0.210	
Localized		
General		
Dental treatment before radiotherapy		
Endodontic treatment	0.379	
Removal of root remainders	0.636	
Tooth extraction	0.291	
Conserving treatment	0.603	
Cystectomy	0.936	
Healthy teeth remaining after dental rehabilitation	0.158	
Demographic and oncological data		
Age	0.106	
Karnofsky performance status	0.625	
T-stage	0.222	
N-stage	0.040	Significant
Total dose	0.005	Significant
BED2	0.040	Significant
Daily fraction	0.036	Significant
**Multivariate analysis**		
Carious teeth	0.0089	Significant
N-stage	0.1256	
Total dose	0.3524	
BED 2	0.4182	
Daily fraction	0.5596	

All remaining factors as mentioned in Tables 1 and 2 have been tested univariately and found insignificant, thus they were not tested multivariately.

We did not try to compare the oncological data like local and regional tumor outcome or survival because it seemed impossible to achieve reliable data for group B nearly 20 years after treatment. Thus, we could not correlate the frequency of IORN to a local recurrence.

## Discussion

### Dental health status and dental rehabilitation procedures

From our data we can summarize that – despite all effort in dental prophylaxis – the dental status of patients with oral neoplasms did not improve” over decades nor did the extent of dental rehabilitation procedures necessary before the start of radiotherapy.

The comparison of our data to those of the Forth German Trial of Oral Health (Kern et al. 2006) resulted in marked differences: In this study adults (33-44 years of age) on average 14.5 teeth were found carious, in older people (> = 45 years of age) 22.1 teeth. These teeth were rehabilitated completely in 95.6% and in 94.8%, respectively. A mean of 2.77 teeth in adults and of 14.2 teeth in older people were missing. 72% of the adults and 60.6% of the seniors were found to perform sufficient mouth hygiene. All these values were improved compared to the results of a former trial in 1997. On the other hand, the frequency of periodontitis was rising (moderate in 52.9% and intense in 39.8% of the population). Compared to those data our findings in patients with oral neoplasms were much more unfavourable and did not improve over time.

Further equally detailed analyses were rare. Jham et al.(Jham et al. 2008) reported in 2008 a collective of 207 patients with head and neck cancer with similar dental findings to our investigation detecting periodontal disease in 41%, retained roots in 21%, carious teeth in 12%, and unerupted teeth in 5.8% of their patients, resulting in an IORN rate of 5.5%. Schuurhuis et al. summarized 2011 the data of 185 patients and found oral infectious foci in 75%, a periodontal pocket depth of more than 6 mm in 23%, severe caries in 4%, impacted teeth in 4%, and residual root tips in 3%. Tooth extractions had to be performed in 30% of the patients, a mean of 7.7 teeth had to be removed. Periodontal treatment was performed in 6%. IORN was diagnosed in 11% (Schuurhuis et al. 2011). Further literature data on this topic have been summarized in Table 5. In general, tumor patients frequently showed a noncompliance in routine dental care and daily oral hygiene. Tumor diagnosis did not change the patients’ habits: Lockhart and Clark stated in 1994 that 97% of their patients needed dental care before radiotherapy, but only 81% underwent the indicated treatment.

**Table 5:** **Dental status and rehabilitation procedures in the literature for IORN in the lower jaw**

Author group	Dental status	Rehabilitation procedures	Remarks
Frydrych and Slack-Smith 2011 (n = 82)	No information	No information	Average (median) date of last dental visit: 66.76 months (18 months) before radiotherapy
Guggenheimer and Hoffman 1994 (n = 947)	Edentulous: 59%	No information	
	Partially edentulous: 9%		
	Poor dentition with no replacement: 14%		
	Intact dentition: 18%		
Maier et al. 1993 (n = 100)	Tumour vs. control patients: Tartar > 3 mm: 40.91 vs. 21.98%	No information	Tumour vs. control patients
			Never tooth brushing 44.9 vs. 23.5%
	Decayed teeth >50% : 27.2 vs. 3.9%		Dental visit more than once a year: 6% vs. 43.5%
Lockhart and Clark 1994 (n = 131)	Alveolar bone loss: 66%	Needing dental care: 97%	Noncompliant with routine dental care: 76%
	Clinical caries: 71%	Did not seek the indicated treatment: 81%	Noncompliant with routine oral hygiene: 65%
	Failing restorations: 91%		
Jham et al. 2008 (n = 207)	Periodontal disease: 41%	No information	
	Residual root: 21.2%		
	Caries 12%		
	Unerupted tooth: 5.8%		

### Frequency of IORN

The frequency of IORN was almost equal in both groups. However, the influence of fractionation was interesting. The rates of IORN were identical after conventional fractionation over the decades. However, while in group B an unacceptably high amount of IORN was diagnosed after hyperfractionation, we did not see any IORN in group A. One reason may be the reduction of the total dose from 82.8 Gy to 72 Gy, another one the extension of the interfraction interval from 4-6 to generally >6 hours, this relevance of interfractional time intervals for cell recovery was not yet known during radiotherapy of group B patients (Fowler, J., personal communication, approx. 1988).

In the literature, the incidence of IORN varied widely (0-74%) as depicted in Table 6 whereas the majority of data are in a range of 5-10%. However, the comparison of these values to each other and to our results is very difficult because of a different definition of staging of IORN, different tumor localizations, therapy schedules, radiation techniques and dosages results fit well within the range of data taken from the literature (Kim et al. 1974; Niewald et al. 1996). One of the data sets in the literature most comparable to our dataset has been published by Lee et al. (Lee et al. 2008) who experienced comparable IORN frequencies in a collective of patients having been operated on mainly.

**Table 6:** **Incidence of IORN of the upper and lower jaw in the literature**

Author group	Incidence	Remarks
Ben-David et al. 2007 (n = 176)	0	Multiple tumour localizations
		Primary treatment (no surgery)
		IMRT
		108/176 radiochemotherapy
Berger and Bensadoun 2010	1-5%	Literature survey
Crombie et al. 2012 (n = 54)	36%	53/54 radiochemotherapy
Gomez et al. 2011 (n = 168)	1.2%	Multiple tumour localizations
		IMRT
Gomez et al. 2009 (n = 35)	5%	IMRT
Jereczek-Fossa and Orecchia 2002	0.4-56%	Literature survey
Jham et al. 2008 (n = 207)	5.5%	Head and neck cancer
Katsura et al. 2008 (n = 39)	15%	
Lee et al. 2008 (n = 189)	6.6%	Oral cavity and oropharynx
Monnier et al. 2011 (n = 73)	40%	Oral cavity and oropharynx
Oh et al. 2004 (n = 81)	4.9%	
Reuther et al. 2003 (n = 830)	8.2%	Oral cavity and oropharynx
Stenson et al. 2010 (n = 27)	18.4%	Surgery, adjuvant radiochemotherapy
Storey et al. 2001 (n = 83)	6%	Malignant submandibular tumours
Studer et al. 2011 (n = 304)	Grade 2 EORTC: 1.6%	Oral cavity and oropharynx
		Conventional dental care vs. risk-adapted dental care
		IMRT
Thiel 1989	4-35%	Literature survey
Thorn et al. 2000 (n = 80)	74%/3 years	Multiple tumour localizations
Tsai et al. 2012 (n = 402)	7.5%	Oropharyngeal cancer, median time to IORN 8 months
Turner et al. 1996 (n = 333)	5.9%	

### Risk factors for the occurrence of IORN

Numerous prognostic factors for the development of IORN have been tested and published. A selection of these is summarized in Table 7. The localization of the primary tumor in the oral cavity with its microbial colonization and the abundant involvement of the mandibular bone with its unique blood supply probably promotes IORN. Unfavorable dental status, periodontal disease and soreness of the gingiva by pressure triggered by dental prosthesis are important as well as dental extractions before and especially after radiotherapy.

**Table 7:** **Risk factors for IORN of the upper and lower jaw in the literature**

Author group	Risk factor(s)	Remarks
Ahmed et al. 2009	Intensity modulated radiotherapy (IMRT) advantageous compared to conventional radiotherapy	
Berger and Bensadoun 2010	Total dose >66 Gy	Literature survey
Bhide et al. 2012	Total dose > 60 Gy	Literature survey
	Volume of mandible within the treatment field. Trauma related ORN after lower doses	
	IMRT	
Chopra et al. 2011	White ethnicity	
	Secondary infection	
	Advanced age	
	Stage IV	
	Total dose	
	Post-RT dental extractions	
	Lack of pre-RT dental extractions	
Curi and Dib 1997, 2007	Oral cancer	
	Invasion of bone	
	Tumour surgery	
	Total radiation dose	
	Dose rate/day	
	Mode of radiation delivery	
	Dental status	
	Time from radiation therapy until the onset of ORN	
Goldwasser et al. 2007	Higher body mass index	Multivariate analysis
	Use of steroids	
	Radiation dose >66 Gy	
Jereczek-Fossa and Orecchia 2002	Total dose	Literature survey, only part of the factions mentioned in the paper cited here
	Brachytherapy dose	
	Dose per fraction	
	Interval between fractions	
	Volume of the horizontal ramus of the mandible irradiated with a high dose	
	Dental status	
	Bad oral hygiene	
	Dental extractions after radiotherapy	
Katsura et al. 2008	Oral health status after radiotherapy	
	Periodontal pocket depth	
	Dental plaque	
	Alveolar bone loss level	
	Radiographic periodontal status	
Lee et al. 2009	Univariate: Mandibular surgery	Multivariate analysis:
	Co-60	Mandibular surgery
		BED >106.2 Gy
Lozza et al. 1997	Dose rate	Brachytherapy exclusively
	Reference volume	
Monnier et al. 2011	Oral cavity tumours	Multivariate analysis: bone surgery
	Bone invasion	
	Surgery prior to radiotherapy	
	Bone surgery	
Nabil 2012	Hyperfractionation	Literature survey
	Reduced risk after accelerated radiotherapy with reduced dose	
Reuther et al. 2003	Advanced tumours	
	Segmental resection of the mandible	
	Tooth extractions (pre/post RT)	
	Pre-surgical radiotherapy worse than post-surgical radiotherapy	
Store and Boysen 2000	Tumour localization in tongue and floor of mouth	
	Trauma	
Thiel 1989	Caries	
	Periondontosis	
	Periapical pathology	
	Injury	
	Irritation by prostheses	
	Dental extractions before and after radiotherapy	
	Bone surgery because of remaining or recurrent tumours	
Thorn et al. 2000	Removal of teeth	
	Surgery	
	Injury from prosthesis	
	Spontaneous breakdowns	
Tsai et al. 2012	Total dose	
	Dental status	
	Smokers	
	Alcohol	
	Larger tumours	
Turner et al. 1996	Bone involvement	
	Synchronous Methotrexate	
	Scattered dose from elective neck treatment	
	Increasing dose	
	Increasing target volumes for doses <55 Gy	
	Dental extractions	

Radiation dose should not exceed 60 – 66 Gy to the mandibular bone whenever possible, the target volume extending to the bone should be limited. Some authors regard hyperfractionation as a risk factor for IORN. In our ancient publication on this topic (Niewald et al. 1996) we experienced a very high frequency of IORN after hyperfractionated radiotherapy which may have been caused by too high total doses on the one hand and a too short interfraction interval (time interval between the two daily fractions) on the other hand. Both factors have been taken into account since 1992, consequently the results were improved markedly.

Intensity modulated radiotherapy (IMRT) has been found advantageous compared to conventional 3D-planned radiotherapy. Additional factors may be chemotherapy, higher body mass index and the use of steroids.

An important paper has been published by Tsai et al. in 2013 (Tsai et al. 2012). They reviewed the records of patients with small oropharyngeal cancers having undergone radiotherapy or radiochemotherapy. The overall prevalence of IORN was 7.5%, higher doses, use of nicotine and alcohol, dental status as well as more advanced tumors were found significant risk factors for the development of IORN. In contrast to this paper our patients’ primary situation seems more unfavorable: we only examined patients with oral cancer where the whole mandible was within the 100%-isodose, thus we applied even higher doses to a large amount of bone. Furthermore, older techniques have been used; unfortunately, no information about fractionation has been given. Consequently, a higher prevalence of IORN here seems to be explainable.

Unfortunately, we did not succeed in identifying clearly significant independent prognostic factors for the development of IORN. In our patient collective, hyperfractionation seemed to have a protective effect whereas this could not be examined further due to the small number of events. In our dataset the number of carious teeth was found to be the only independent prognostic factor after multivariate analysis. Univariately, total dose and BED2 were significant which could be expected. In majority of patients, the total doses lie in a narrow range of 60-82 Gy which may have been a reason for the result, additionally the fact that few IORN cases have been observed.

The authors are well aware of the limitations of this retrospective evaluation. In this nearly homogenous collective of patients with oral cavity cancer having undergone radiotherapy +/- surgery, we have found complete data sets with respect to the dental status and restoration procedures of nearly all patients. The IORN data have been investigated meticulously, but due to the known incompliance of head and neck patients we could not exclude that single events did not become known to the authors.

## Conclusions

The patients’ dental status before radiotherapy was very poor compared to an otherwise healthy population. Apparently we did not succeed in improving these findings over the decades despite all effort in terms of dental prophylaxis. Consequently, extensive dental rehabilitation procedures had to be performed which did not change over time as well.

Examining patients irradiated with conventional fractionation, the incidence of IORN was found constant in a range of 10-15% over time. As stated earlier, the influence of the interfraction interval and of very high doses became known after the patients in group B had been irradiated, we thus diagnosed an unacceptably high frequency of IORN which became virtually zero after reduction of the total dose and extension of the interfraction interval. The multivariate search for prognostic factors only resulted in the assumption that dental status and fractionation could influence the occurrence of IORN.

## Abbreviations

BED2: Biologically effective dose (α/β = 2 Gy)
DEGRO: German society for radiation oncology
IMRT: Intensity modulated radiotherapy
IORN: Infected osteoradionecrosis
ORN: Osteoradionecrosis
UICC: Union internationale contre le cancer.

## References

[CR1] Ahmed M, Hansen VN, Harrington KJ, Nutting CM (2009). Reducing the risk of xerostomia and mandibular osteoradionecrosis: the potential benefits of intensity modulated radiotherapy in advanced oral cavity carcinoma. Med Dosim.

[CR2] Barbie O (1997). Risikofaktoren und Dosis-Effekt-Beziehungen der Osteoradionekrose nach hyperfraktionierter und konventionell fraktionierter Strahlentherapie von Mundhöhlenkarzinomen – eine retrospektive Studie.

[CR3] Ben-David MA, Diamante M, Radawski JD, Vineberg KA, Stroup C, Murdoch-Kinch CA, Zwetchkenbaum SR, Eisbruch A (2007). Lack of osteoradionecrosis of the mandible after intensity-modulated radiotherapy for head and neck cancer: likely contributions of both dental care and improved dose distributions. Int J Radiat Oncol Biol Phys.

[CR4] Berger A, Bensadoun RJ (2010). [Normal tissue tolerance to external beam radiation therapy: the mandible]. Cancer Radiother.

[CR5] Bhide SA, Ahmed M, Newbold K, Harrington KJ, Nutting CM (2012). The role of intensity modulated radiotherapy in advanced oral cavity carcinoma. J Cancer Res Ther.

[CR6] Chopra S, Kamdar D, Ugur OE, Chen G, Peshek B, Marunick M, Kim H, Lin HS, Jacobs J (2011). Factors predictive of severity of osteoradionecrosis of the mandible. Head Neck.

[CR7] Crombie AK, Farah C, Tripcony L, Dickie G, Batstone MD (2012). Primary chemoradiotherapy for oral cavity squamous cell carcinoma. Oral Oncol.

[CR8] Curi MM, Dib LL (1997). Osteoradionecrosis of the jaws: a retrospective study of the background factors and treatment in 104 cases. J Oral Maxillofac Surg.

[CR9] Curi MM, Oliveira dos Santos M, Feher O, Faria JC, Rodrigues ML, Kowalski LP (2007). Management of extensive osteoradionecrosis of the mandible with radical resection and immediate microvascular reconstruction. J Oral Maxillofac Surg.

[CR10] Frydrych AM, Slack-Smith LM (2011). Dental attendance of oral and oropharyngeal cancer patients in a public hospital in Western Australia. Aust Dent J.

[CR11] Goldwasser BA, Chuang S-K, Kabal LB, August M (2007). Risk factor assessment for the development of osteoradionecrosis. J Oral Maxillofac Surg.

[CR12] Gomez DR, Zhung JE, Gomez J, Chan K, Wu AJ, Wolden SL, Pfister DG, Shaha A, Shah JP, Kraus DH, Wong RJ, Lee NY (2009). Intensity-modulated radiotherapy in postoperative treatment of oral cavity cancers. Int J Radiat Oncol Biol Phys.

[CR13] Gomez DR, Estilo CL, Wolden SL, Zelefsky MJ, Kraus DH, Wong RJ, Shaha AR, Shah JP, Mechalakos JG, Lee NY (2011). Correlation of osteoradionecrosis and dental events with dosimetric parameters in intensity-modulated radiation therapy for head-and-neck cancer. Int J Radiat Oncol Biol Phys.

[CR14] Guggenheimer J, Hoffman RD (1994). The importance of screening edentulous patients for oral cancer. J Prosthet Dent.

[CR15] Jereczek-Fossa BA, Orecchia R (2002). Radiotherapy-induced mandibular bone complications. Cancer Treat Rev.

[CR16] Jham BC, Reis PM, Miranda EL, Lopes RC, Carvalho AL, Scheper MA, Freire AR (2008). Oral health status of 207 head and neck cancer patients before, during and after radiotherapy. Clin Oral Investig.

[CR17] Katsura K, Sasai K, Sato K, Saito M, Hoshina H, Hayashi T (2008). Relationship between oral health status and development of osteoradionecrosis of the mandible: a retrospective longitudinal study. Oral Surg Oral Med Oral Pathol Oral Radiol Endod.

[CR18] Kern R, Krämer J, Michaelis W (2006). Vierte Deutsch Mundgesundheitsstudie des Instituts der Deutschen Zahnärzte.

[CR19] Kim JH, Chu FC, Pope RA, Woodard HQ, Bragg DB, Shidnia H (1974). Proceedings: time dose factors in radiation induced osteitis. Am J Roentgenol Radium Ther Nucl Med.

[CR20] Lee JA, Huh SJ, Oh D, Bae DS (2008). Osteoradionecrosis after three-dimensional conformal radiotherapy for recurrent cervical cancer presenting as a progressive osteolytic lesion. Ann Nucl Med.

[CR21] Lee IJ, Koom WS, Lee CG, Kim YB, Yoo SW, Keum KC, Kim GE, Choi EC, Cha IH (2009). Risk factors and dose-effect relationship for mandibular osteoradionecrosis in oral and oropharyngeal cancer patients. Int J Radiat Oncol Biol Phys.

[CR22] Lockhart PB, Clark J (1994). Pretherapy dental status of patients with malignant conditions of the head and neck. Oral Surg Oral Med Oral Pathol.

[CR23] Lozza L, Cerrotta A, Gardani G, De Marie M, Di Russo A, Kenda R, Tana S, Valvo F, Zucali R (1997). Analysis of risk factors for mandibular bone radionecrosis after exclusive low dose-rate brachytherapy for oral cancer. Radiother Oncol.

[CR24] Maier H, Zoller J, Herrmann A, Kreiss M, Heller WD (1993). Dental status and oral hygiene in patients with head and neck cancer. Otolaryngol Head Neck Surg.

[CR25] Mang C (2011). Retrospektive Untersuchung zur Häufigkeit und Risikofaktoren einer infizierten Radioosteonekrose bei Radiotherapie von Mundhöhlenkarzinomen.

[CR26] Monnier Y, Broome M, Betz M, Bouferrache K, Ozsahin M, Jaques B (2011). Mandibular osteoradionecrosis in squamous cell carcinoma of the oral cavity and oropharynx: incidence and risk factors. Otolaryngol Head Neck Surg.

[CR27] Nabil S (2012). Redefining osteoradionecrosis. Oral Surg Oral Med Oral Pathol Oral Radiol.

[CR28] Niewald M, Barbie O, Schnabel K, Engel M, Schedler M, Nieder C, Berberich W (1996). Risk factors and dose-effect relationship for osteoradionecrosis after hyperfractionated and conventionally fractionated radiotherapy for oral cancer. Br J Radiol.

[CR29] Niewald M, Fleckenstein J, Mang K, Holtmann H, Spitzer WJ, Rube C (2013). Dental status, dental rehabilitation procedures, demographic and oncological data as potential risk factors for infected osteoradionecrosis of the lower jaw after radiotherapy for oral neoplasms: a retrospective evaluation. Radiat Oncol.

[CR30] Oh HK, Chambers MS, Garden AS, Wong PF, Martin JW (2004). Risk of osteoradionecrosis after extraction of impacted third molars in irradiated head and neck cancer patients. J Oral Maxillofac Surg.

[CR31] Reuther T, Schuster T, Mende U, Kubler A (2003). Osteoradionecrosis of the jaws as a side effect of radiotherapy of head and neck tumour patients--a report of a thirty year retrospective review. Int J Oral Maxillofac Surg.

[CR32] Schuurhuis JM, Stokman MA, Roodenburg JL, Reintsema H, Langendijk JA, Vissink A, Spijkervet FK (2011). Efficacy of routine pre-radiation dental screening and dental follow-up in head and neck oncology patients on intermediate and late radiation effects. A retrospective evaluation. Radiother Oncol.

[CR33] Schwartz HC, Kagan AR (2002). Osteoradionecrosis of the mandible: scientific basis for clinical staging. Am J Clin Oncol.

[CR34] Stenson KM, Kunnavakkam R, Cohen EE, Portugal LD, Blair E, Haraf DJ, Salama J, Vokes EE (2010). Chemoradiation for patients with advanced oral cavity cancer. Laryngoscope.

[CR35] Store G, Boysen M (2000). Mandibular osteoradionecrosis: clinical behaviour and diagnostic aspects. Clin Otolaryngol Allied Sci.

[CR36] Storey MR, Garden AS, Morrison WH, Eicher SA, Schechter NR, Ang KK (2001). Postoperative radiotherapy for malignant tumors of the submandibular gland. Int J Radiat Oncol Biol Phys.

[CR37] Studer G, Glanzmann C, Studer SP, Gratz KW, Bredell M, Locher M, Lutolf UM, Zwahlen RA (2011). Risk-adapted dental care prior to intensity-modulated radiotherapy (IMRT). Schweiz Monatsschr Zahnmed.

[CR38] Thiel HJ (1989). Osteoradionecrosis. I. Etiology, pathogenesis, clinical aspects and risk factors. Radiobiol Radiother (Berl).

[CR39] Thorn JJ, Hansen HS, Specht L, Bastholt L (2000). Osteoradionecrosis of the jaws: clinical characteristics and relation to the field of irradiation. J Oral Maxillofac Surg.

[CR40] Tsai CJ, Hofstede TM, Sturgis EM, Garden AS, Lindberg ME, Wei Q, Tucker SL, Dong L (2012). Osteoradionecrosis and radiation dose to the mandible in patients with oropharyngeal cancer. Int J Radiat Oncol Biol Phys.

[CR41] Turner SL, Slevin NJ, Gupta NK, Swindell R (1996). Radical external beam radiotherapy for 333 squamous carcinomas of the oral cavity--evaluation of late morbidity and a watch policy for the clinically negative neck. Radiother Oncol.

